# The treatment landscape in axial spondyloarthritis—do we need new therapies?

**DOI:** 10.1016/j.ero.2025.11.020

**Published:** 2025-12-12

**Authors:** Denis Poddubnyy

**Affiliations:** 1 Division of Rheumatology, University of Toronto, Toronto, ON, Canada; 2 Schroeder Arthritis Institute, University Health Network, Toronto, ON, Canada; 3 Department of Gastroenterology, Infectiology and Rheumatology (including Nutrition Medicine), Charité-Universitätsmedizin Berlin, Berlin, Germany

## Abstract

Despite major therapeutic advances, many patients with axial spondyloarthritis (axSpA) fail to achieve remission or even low disease activity with currently available advanced therapies. This highlights an urgent need to understand why treatment responses remain suboptimal and how management can be improved. Several mechanisms contribute to persistent symptoms. Misdiagnosis and overdiagnosis remain frequent, often due to nonspecific imaging findings being misattributed to axSpA. Among correctly diagnosed patients, a substantial proportion experience pain not directly driven by inflammation. Nociplastic pain—frequently associated with fatigue, anxiety, depression, and sleep disturbances—is the predominant mechanism, while neuropathic pain is less common. These patients do not benefit from escalation of anti-inflammatory therapy but instead require tailored approaches addressing nociplastic pain, including structured exercise, cognitive-behavioural therapy, and pharmacological agents. However, robust clinical trial data in axSpA populations are lacking, and studies evaluating these interventions as add-on strategies are urgently needed. A smaller but clinically important subset of patients demonstrates true treatment-refractory (TR) axSpA, with objective inflammation persisting despite multiple biologic or targeted synthetic disease-modifying antirheumatic drugs. Novel therapeutic approaches under investigation include dual-targeted therapy with complementary mechanisms, selective depletion of pathogenic T cells, modulation of the HLA-B27 immunopeptidome through endoplasmic reticulum aminopeptidase 1/2 inhibition, and blockade of macrophage migration inhibitory factor. The primary challenge in axSpA today—besides the problem of the correct diagnosis—is to optimise the use of existing therapies for pain not related to inflammation, while simultaneously developing innovative options for TR disease. Together, these efforts will determine whether remission can become an attainable goal for all patients.

## INTRODUCTION

Axial spondyloarthritis (axSpA) is a chronic immune-mediated inflammatory disease primarily affecting the spine and sacroiliac joints [1]. It encompasses both radiographic axSpA (formerly termed ankylosing spondylitis) and nonradiographic axSpA [2], distinguished by the presence or absence of definite sacroiliitis on conventional radiographs. The overarching goal of treatment, as emphasised in the treat-to-target recommendations for spondyloarthritis, is to achieve remission, defined as the absence of clinical and laboratory signs of active disease [3]. The Assessment of SpondyloArthritis international Society (ASAS) and the European Alliance of Associations for Rheumatology management recommendations advocate a treat-to-target strategy, aiming for remission or, when remission is not achievable, sustained low disease activity through structured monitoring and timely adaptation of therapy [4]. Despite the availability of several effective pharmacological options, a substantial proportion of patients with axSpA do not achieve remission or low disease activity [5]. This persistent therapeutic gap highlights the need both to refine existing management strategies and to develop novel approaches for patients whose symptoms remain uncontrolled despite effective anti-inflammatory therapy.

### Current treatment options

The management of axSpA is based on a combination of nonpharmacological and pharmacological interventions. Pharmacological treatment begins with nonsteroidal anti-inflammatory drugs (NSAIDs), which remain the first-line therapy for pain and stiffness. If disease activity remains high despite optimal use of NSAIDs, escalation to advanced therapies is warranted [4].

Advanced therapies with proven efficacy in axSpA include biologic disease-modifying antirheumatic drugs (DMARDs), specifically tumour necrosis factor inhibitors and interleukin (IL)-17 inhibitors (targeting IL-17A or IL-17A/F), as well as targeted synthetic DMARDs, namely Janus kinase inhibitors. Although head-to-head randomised controlled trials directly comparing drug classes in axSpA are largely lacking, the open-label SURPASS study compared secukinumab with an adalimumab biosimilar regarding their ability to inhibit structural damage progression in the spine. The trial demonstrated comparable radiographic outcomes and similar clinical responses between both treatments, with slightly higher response rates for adalimumab in outcomes incorporating C-reactive protein [6]. Phase 3 clinical trials have consistently shown broadly comparable results concerning musculoskeletal outcomes (although extramusculoskeletal manifestations—anterior uveitis, psoriasis, and inflammatory bowel disease—respond differently to various drug classes, which often determines treatment choice in clinical practice [4]), with approximately 40%-50% of patients achieving an ASAS 40% response (ASAS40) and remission rates being considerably lower [7]. This raises the central question: why do current therapeutic strategies fail to deliver higher response and remission rates despite major advances in the treatment of rheumatic diseases over recent decades?

### Challenges in treatment nonresponse

Even with optimal use of advanced therapies, a substantial proportion of patients with axSpA continue to experience symptoms—most prominently pain—that limit quality of life. Several factors contribute to this apparent nonresponse.

A frequent issue is misdiagnosis or overdiagnosis, often related to the interpretation of imaging [8]. Nonspecific bone marrow oedema on magnetic resonance imaging (MRI) of the sacroiliac joints, resulting from mechanical or degenerative changes, may be misattributed to axSpA [9]. As a consequence, some patients receive biologic therapy without having a true inflammatory disease, and treatment inevitably appears ineffective. This issue is most relevant to clinical practice, where MRI interpretation is performed locally. In clinical trials, central reading of imaging reduces the likelihood of misclassification, although it cannot fully prevent it, as central readers base their assessments solely on imaging findings without access to the full clinical context.

In patients with confirmed axSpA, persistent symptoms may also reflect nociplastic pain mechanisms such as central sensitisation (with fibromyalgia representing the extreme phenotype of this spectrum), often accompanied by fatigue, anxiety, depression, and sleep disturbances [[10], [11], [12], [13]]. Female sex and the presence of obesity are factors increasing the likelihood of nociplastic pain and may therefore contribute to nonresponse [13,14]. Patients with nociplastic pain report ongoing pain despite well-controlled inflammation [15] and do not benefit from escalation of anti-inflammatory therapy.

Another non-nociceptive pain mechanism, neuropathic pain, can be present but is relatively rare as the predominant driver in axSpA [15]. As a result of the presence of nociplastic and neuropathic pain—pain mechanisms that cannot be directly influenced by anti-inflammatory drugs—patient-reported outcome-based measures of response (including the commonly used primary endpoint ASAS40) may indicate apparent nonresponse even to the most potent anti-inflammatory treatment.

Finally, a small subset of patients experience true treatment-refractory (TR) axSpA, defined by objective evidence of inflammation (eg, elevated C-reactive protein or active inflammation on MRI) persisting despite the use of multiple biologic or targeted synthetic DMARDs.

To capture the full spectrum of cases with persistent problems despite effective anti-inflammatory treatment, ASAS has proposed the concept of difficult-to-manage (D2M) axSpA, which includes TR disease [16]. This construct encompasses patients with ongoing disease burden despite guideline-based therapy and differentiates between D2M and TR cases. First published real-world data suggest that about 10% of patients meet criteria for D2M axSpA, whereas only 1% to 2% fulfil the stricter TR definition [17]. Prospective studies should disentangle the exact mechanisms underlying D2M and TR disease to optimise management strategies and inform future research.

Together, these scenarios illustrate that although many cases of nonresponse are attributable to diagnostic uncertainty or noninflammatory pain mechanisms, true refractory disease is uncommon. At the same time, both scenarios represent a clear unmet need in clinical practice and research.

### Future directions in therapy—do we need new therapies?

#### Proper patient selection: avoiding misdiagnosis and misclassification in clinical practice and research

Accurate patient selection remains a critical prerequisite for effective disease management and meaningful interpretation of treatment outcomes in axSpA [8]. The limited availability of reliable diagnostic biomarkers and the nonspecific nature of the cardinal clinical manifestation of axSpA—back pain—continues to pose a major challenge, making the integration of clinical assessment, imaging, and laboratory findings essential.

Proper recognition of inflammatory changes caused by immune-mediated inflammation requires careful interpretation of imaging findings in the context of the patient’s clinical presentation. Inflammatory lesions on MRI in axSpA typically localise to the subchondral bone marrow of the middle portion of the cartilaginous compartment of the sacroiliac joint, often accompanied by structural lesions such as erosions, backfill, or fat metaplasia. In contrast, nonspecific or mechanically induced bone marrow oedema (eg, in osteitis condensans ilii) is usually located in the anterior or inferior joint portions, frequently associated with sclerosis and absence of erosions. Recognition of these typical patterns, together with comprehensive clinical information and adequate communication between rheumatologists and radiologists, is essential to reduce diagnostic error and avoid misclassification [9].

Ongoing research focuses on improving the detection and differentiation of immune-mediated inflammation through advanced imaging and imaging interpretation. Promising directions include artificial-intelligence-assisted image reading and molecular imaging modalities such as positron emission tomography targeting cytokines or immune-cell subsets involved in spondyloarthritis pathogenesis.

#### Addressing symptoms not related to inflammation: non-nociceptive pain

Multidisciplinary approaches are central to the management of nociplastic pain (including patients with secondary fibromyalgia) and should include physiotherapy, structured exercise, and psychological interventions such as cognitive-behavioural therapy where appropriate. Identifying and addressing underlying contributors—including depression, anxiety, and sleep disorders—are essential to tailoring an effective treatment strategy [18].

Pharmacological options for nociplastic pain comprise serotonin-norepinephrine reuptake inhibitors such as duloxetine, low-dose tricyclic antidepressants (eg, amitriptyline), gabapentinoids (eg, pregabalin), and, the muscle relaxant cyclobenzaprine, which has been approved recently for fibromyalgia [[18], [19], [20]]. Neuropathic pain, though less frequent as a primary mechanism in axSpA, can also be targeted pharmacologically in selected patients.

Importantly, most of the available evidence for these pharmacological interventions comes from studies in fibromyalgia or chronic pain cohorts without inflammatory rheumatic disease. Robust clinical trials in axSpA are lacking. In the author’s view, systematic studies evaluating these therapies as add-on strategies to anti-inflammatory treatment are urgently needed to better define their role in clinical practice.

#### Novel approaches for TR axSpA: is there something new on the horizon?

For the small subset of patients with TR axSpA, defined by persistent objective inflammation despite use of multiple advanced therapies, there remains a clear unmet need for novel strategies. The recently proposed ASAS definitions of D2M and TR axSpA [16] are expected to stimulate research that will help clarify the mechanisms underlying inflammation unresponsive to currently available advanced treatments.

From a therapeutic perspective, one area of exploration is the combination of advanced therapies. Although evidence is limited, early reports suggest that combining agents with complementary mechanisms may improve outcomes in selected patients [[21], [22], [23]]. However, robust data from controlled clinical trials are still scarce, and questions remain regarding long-term safety and feasibility.

Beyond combination approaches, mechanism-based innovations are emerging. Targeting pathogenic CD8⁺ T-cell subsets—particularly TRBV9⁺ T cells that recognise HLA-B27–restricted peptides—represents one promising strategy. Selective depletion of TRBV9⁺ T cells is under active investigation [24]; future studies will show whether other, potentially more targeted, approaches for pathogenic T-cell elimination can be developed. Another avenue is modulation of the immunopeptidome by inhibiting endoplasmic reticulum aminopeptidases 1/2, thereby altering the peptide repertoire presented by HLA-B27 and potentially preventing the activation of autoreactive T cells [25].

Further, macrophage migration inhibitory factor (MIF) has been identified as an upstream regulator of Th-17 pathways and has shown strong pathogenic relevance in preclinical models [26]. Inhibiting MIF may therefore represent an additional therapeutic strategy in patients with refractory disease.

## CONCLUSIONS

Despite substantial progress in the management of axSpA, a significant proportion of patients continue to experience symptoms, particularly pain, which negatively impacts quality of life. A key challenge that precedes and contributes to apparent treatment failure is diagnostic inaccuracy, leading to initiation or escalation of therapy in patients whose symptoms are not primarily driven by immune-mediated inflammation. The most pressing need in daily practice for patients with definite axSpA—given its relatively high frequency compared to true biological nonresponse—is to optimise care for patients whose symptoms are not primarily inflammation driven. This requires structured application of available pharmacological and nonpharmacological interventions, ideally supported by clinical trial evidence specifically generated in axSpA. At the same time, a smaller but clinically important group with true TR disease continues to require innovative therapeutic strategies.

‘Do we need new therapies?’ The likely future answer is that new therapies, together with optimised use of established ones, will help make remission an attainable goal for every patient with axSpA.

## Funding

Supported by Vienna Medical Academy.

## Competing interests

DP reports a relationship with AbbVie, Bristol Myers Squibb, Eli Lilly, Johnson and Johnson, Novartis, Pfizer, UCB that includes: funding grants; reports a relationship with AbbVie, Eli Lilly, GlaxoSmithKline, Greywolf Therapeutics, Johnson and Johnson, Merk, Moonlake, Novartis, Pfizer, and UCB that includes: consulting or advisory; reports a relationship with AbbVie, Canon, Celltrion, Eli Lilly, Globemed, Johnson and Johnson, Medscape, Novartis, Peervoice, Pfizer, and UCB that includes: speaking and lecture fees; and reports a relationship with ASAS that includes: board membership.

## Patient consent for publication

Not applicable.

## Ethics approval

Not applicable.

## Provenance and peer review

Commissioned; externally peer reviewed.

## CRediT authorship contribution statement

**Denis Poddubnyy:** Writing – original draft, Methodology, Conceptualization.
